# Wise Charities

**Published:** 1860-04

**Authors:** 


### WISE CHARITIES.

LIFE ILLUSTRATED, ever alive to the diffusion of intelligence which its editors believe tends to ameliorate the condition of the suffering, the friendless, and the poor, says that Alfred White, clerk of St. Paul's Mission, has established in Laight street a "Church Home Intelligence Office," where unprotected females, of good character, may find an asylum while unemployed.

Also, that there is at least one hotel in the city of New-York where a sick man can secure some little attention, and even nursing, without its costing him ten dollars a day, for the mere shadow of these things. In the bare hope that it may be a "Maison Sante," a Home of Health, we give its locality, No. 153 Bleecker street. We once took a friend to an up-town boarding-house. He was very ill. His bill for "accommodations" for four days was eighty dollars, while we did the nursing.

Another of the deserving charities, and of rather a novel character, is referred to in the *Express* by its editor, the Hon. Erastus Brooks, a large portion of whose time is expended in personal attention to the eleemosynaries of the city. He states that at No. 31 Vesey street twenty-five tickets can be purchased for a dollar, each one of which will give a poor outcast, a friendless boy or girl or woman, a comfortable night's lodging or a good meal of victuals.

Who of our readers will smoke a cigar after this, and yet profess that he can not afford to give any thing to support a charity of this kind? The price of a cigar will give a bed for a night to a human brother or sister, who else would have to sleep in a barrel, or stoop, or store-box, or be taken to prison; not only a bed, but a sufficient breakfast after it, enough to give strength to set out in the morning and hunt for a job or a home. Pampered young ladies of New-York, look on your diamond rings, your breast-pins of costliest material, your bracelets of solid gold, and remembering how many famishing women and children, and discouraged men, the price of these would comfort and cheer, and mayhap save from discouragement and crime, look at them, and rather than say you have nothing to give, sell them for what they will bring, and deposit the proceeds with Mr. Whitfield for tickets, which carry in your pocket, and when you meet a brother or sister famishing for bread; or when you are importuned by some pallid child or gray-haired woman, "For the love of God, a penny to buy a bit of bread," give a ticket; and it will show where a full meal can be had for it. And as disease follows close on to the heels of destitution, you may thus keep many well. To show that such institutions are needed, nineteen hundred meals and lodgings were supplied at four cents each at three hundred and sixty-seven Pearl street, New-York, in one month. A similar establishment is found at Eighty-one Third avenue, corner of Twelfth street. No doubt George F. Cooledge has a hand in these matters. Such of our readers as have a sympathy for the poor and money to ventilate it, may feel assured that what they give in this direction will be expended wisely and to the best advantage, while the money given to street beggars encourages vagrancy, and is very often spent foolishly if not criminally.

